# Phosphorus limitation heightens vulnerability of *Crocosphaera watsonii* to ocean warming compared with iron limitation

**DOI:** 10.3389/fmicb.2025.1718897

**Published:** 2026-01-06

**Authors:** Amjad A. Mansour, Laura M. Gholmieh, Ellya C. Gholmieh, Ran Duan, Xiaopeng Bian, Yutong Chen, Seth G. John, David A. Hutchins, Fei-Xue Fu

**Affiliations:** Marine and Environmental Sciences, Department of Biological Sciences, University of Southern California, Los Angeles, CA, United States

**Keywords:** *Crocosphaera*, nitrogen fixation, nutrient limitation, temperature, iron-limitation, phosphorus-limitation, ocean warming

## Abstract

**Introduction:**

Phytoplankton rely on diazotrophs like the globally important *Crocosphaera watsonii* to provide bioavailable nitrogen through nitrogen fixation. Given predicted global warming and oligotrophic scenarios, we investigated how nutrient limitation modulates *C. watsonii*’s response to ocean warming, to better understand future marine ecosystem health.

**Methods:**

We performed complete temperature curves (20–34 °C) under three nutrient conditions [iron (Fe)-limited, phosphorus (P)-limited, and Fe/P-replete] to compare physiological responses and test previously hypothesized temperature-nutrient interactions.

**Results:**

Across the viable temperature range, replete culture growth and fixation rates showed narrower, unimodal-like curves, contrasting the greater plateauing witnessed in nutrient-limited thermal curves. Under both limitations, nitrogen fixation was more impacted than carbon fixation. Fe-limited cultures performed better at higher temperatures (survival range: 22–34 °C) and shared thermal optima with replete cultures for growth rates (28–32 °C), while P-limited cultures performed better at lower temperatures (survival range: 20–32 °C) with a narrower optimum range (28–30 °C). Furthermore, extreme temperatures appear to outweigh and override nutrient limitation effects at the warmer end for Fe-limited cultures (34 °C) and the cooler end for P-limited cultures (20 °C), as no significant differences were observed between limited and replete cultures for both growth and fixation rates at these temperatures.

**Discussion:**

Given predicted sea temperature increases and the rising frequency and intensity of oceanic heat waves, our results suggest that *C. watsonii* in P-limited regimes like the North Atlantic may be more vulnerable to warming than in Fe-limited regimes like the North Pacific. P-limitation may force *C. watsonii* to migrate to higher, cooler latitudes for survival, possibly leaving lower-latitude phytoplankton and ecosystems more vulnerable to nitrogen limitation.

## Introduction

Phytoplankton photosynthesis accounts for nearly half of the earth’s primary productivity. However, in the oligotrophic ocean gyres, their growth is constrained by limited amounts of bioavailable nitrogen (N) (Behrenfeld et al., 2006). Phytoplankton and other marine life thus depend on nitrogen fixation (N_2_-fixation) by diazotrophs such as *Crocosphaera watsonii* to fix dissolved, inert dinitrogen (N_2_) gas in ocean surfaces into the bioavailable form of ammonia (NH_3_) (Yang et al., 2021). Besides their role in the N_2_-cycle, diazotrophs further bolster the global carbon cycle by supporting primary productivity, decreasing atmospheric carbon dioxide (CO_2_) and recycling carbon into the ocean through carbon fixation (CO_2_-fixation), fueling the ocean biological carbon pump (Karl et al., 1997).

Of the three main types of marine planktonic diazotrophic cyanobacteria, *C. watsonii* belongs to the unicellular cyanobacteria (UCYN) group and is classified specifically as UCYN-B (Luo et al., 2012). The various UCYN clades represent the most recently discovered and least taxonomically resolved groups and were thus the least studied historically (Zehr et al., 2001; Mareš et al., 2019). While other types of diazotrophs like *Trichodesmium,* which belongs to the non-heterocystous filamentous cyanobacteria group, were historically considered the main contributors of N_2_-fixation, field measurements have highlighted the comparably high fixation rates by UCYN (Montoya et al., 2004), with some studies estimating that UCYN may contribute ~50% of global oceanic N_2_-fixation (Monteiro et al., 2010).

Current research continues to support this paradigm shift, with additional evidence for *C. watsonii*’s specific role. A 2025 study suggests *C. watsonii* (UCYN-B) may dominate and drive high N_2_-fixation rates in certain hotspots of the global ocean, particularly in the (sub)tropical western North Pacific (Jiang et al., 2025). Thus, UCYN-B may serve as a larger contributor to global N_2_-fixation than previously considered, especially in areas where its ecological niche interacts with varying temperature, iron (Fe), and phosphorus levels (P) (Inomura, 2025). Understanding *C. watsonii*’s physiology and growth in the face of predicted climate change scenarios and nutrient availability is therefore an important step toward understanding the fate of primary productivity in the future ocean, especially in nitrogen-limited areas.

Previous studies have suggested that *C. watsonii’s* growth and N_2_-fixation rates are impacted by temperature, nutrient availability, or a combination of the two (Fu et al., 2014; Deng et al., 2021; Yang et al., 2021). Compared to the 1995–2014 baseline average, the Intergovernmental Panel on Climate Change (IPCC) SSP5-8.5 scenario predicts a gradual anthropogenic warming of global sea surface temperatures by approximately 2.01–4.07 °C by the year 2,100 (Fox-Kemper et al., 2021). Increasing temperatures have been shown to increase growth and N_2_-fixation rates up to a thermal optimum in *C. watsonii* in a unimodal temperature curve (Deng et al., 2021; Yang et al., 2021). While the diazotroph does appear to have some limited acclimation and adaptation abilities to higher temperatures (Qu et al., 2022), extensive warming beyond its optimum and especially above its maximum thermal limit (>35–36 °C) may lead to its eventual disappearance (Fu et al., 2014). Furthermore, while temperature itself has been shown to impact diazotroph metabolism and N_2_-fixation rates, recent studies suggest a more interactive, rather than additive, relationship between warming oceans and limited nutrient availability, especially with regards to Fe and P (Jiang et al., 2018; Deng et al., 2021; Yang et al., 2021; Hutchins and Tagliabue, 2024).

Fe inputs into open ocean waters are largely atmospheric, often deriving from Asian and Saharan windborne dust. However, much of the vast ocean gyres remains relatively Fe-poor (Mahowald et al., 2005; Hutchins and Boyd, 2016; Letelier et al., 2019). Diazotrophs have high Fe requirements to support photosynthesis and respiration, and especially N_2_-fixation, where it is a catalytic cofactor in the metalloenzyme nitrogenase (Saito et al., 2011). *C. watsonii* is thought to be better suited than other diazotrophs for Fe-limited conditions because it partitions its photosynthesis during the day and N_2_-fixation during the night, shuttling cellular Fe between the enzymes and proteins involved in both processes (Saito et al., 2011; Sohm et al., 2011; Jacq et al., 2014). Furthermore, its small size and, consequently, large surface area to volume ratio gives it an advantage by increasing Fe uptake ability from the environment (Finkel et al., 2009; Inomura et al., 2019a). Additionally, in Fe-limited oceans, warming temperatures may decrease the negative effects of Fe limitation on *C. watsonii* to a certain extent, as higher temperatures may increase the Fe usage efficiency of N_2_-fixation (N-IUE) (mol N fixed hr.^−1^ mol intracellular Fe^−1^) (Jiang et al., 2018; Yang et al., 2021; Hutchins and Tagliabue, 2024). Together, these advantages may allow *C. watsonii* to outcompete other diazotrophs in warming Fe-limited tropical and subtropical gyres.

P is also an essential component for life, present in phospholipids, nucleic acids, and bioenergetic compounds like ATP (Elser, 2012). Oligotrophic ocean surfaces are replenished with P mostly from vertical advection of nutrient-rich deeper waters (Gupta et al., 2022). However, climate projection models using the Coupled Model Intercomparison Project (CMIP) phases 3 and 5 have indicated that increased surface-ocean temperatures will lead to enhanced stratification of ocean layers in the oligotrophic gyres, reducing the vertical flux of nutrients and leading to greater P-limitation (Capotondi et al., 2012; Fu et al., 2016). Furthermore, *C. watsonii* is more disadvantaged by low P availability than other diazotrophs such as *Trichodesmium*, as it is unable to utilize phosphonates, which account for 25% of available organic P in oceans (Clark et al., 1998; Dyhrman and Haley, 2006). Deng et al. (2021) found that higher temperatures may reduce P-limitation effects, leading to reduced differences in the growth rates between P-limited and replete cultures at higher temperatures and higher P-use efficiencies for N_2_-fixation and CO_2_-fixation.

Two prior studies on the effects of temperature and nutrient-limiting conditions on *C. watsonii* have focused only on single nutrient limitations and covered only three temperatures (Deng et al., 2021; Yang et al., 2021). As a result, little is known about how *C. watsonii* fares across the broader temperature spectrum in oligotrophic conditions, especially at the lower end and near its thermal limits. Our research represents an attempt to comprehensively assess the synergistic impacts of elevated temperature and nutrient limitations by either Fe or P over the complete thermal response curve of *C. watsonii*. This design allows us to directly compare how two major oligotrophic nutrient stressors can differentially and exclusively shape *C. watsonii*’s thermal performance, something that had not been possible in earlier, narrower studies. Additionally, determining thermal optima and survival durations at peak temperatures under both types of prevailing nutrient limitation can offer new insights into the future growth, distribution, and modeling of this globally important diazotroph in future changing oceanic conditions, including during extreme marine heat wave events.

## Materials and methods

### Culturing methods

The *C. watsonii* strain WH0005 used in this study was isolated from the North Pacific Ocean (Webb et al., 2009). Three nutrient conditions were included in the experiment: Fe-limited, P-limited, and Fe/P-replete. The cultures in the Fe/P-replete condition (which hereafter will be referred to as “replete”) were well-supplied with both Fe and P, while Fe-limited cultures were well-supplied with P but only limited Fe. Likewise, P-limited cultures were supplied with ample Fe but with limited P. For each of these three nutrient conditions, triplicate cultures were grown across the full thermal range of *C. watsonii,* spanning eight different temperatures (20, 22, 24, 26, 28, 30, 32, 34 °C). Cultures were acclimated to their respective temperature by gradually transferring from the nearest higher or lower temperature cultures exhibiting adequate growth (with ~3–4 days between transfers), starting with the stock culture at 28 °C. Initial cell counts used to run experiments were 4.3–4.7 × 10^4^ cells per mL. Three unsuccessful, independent attempts were made to acclimate and grow P-limited cultures at 18 °C and 34 °C (viable range: 20–32 °C), as well as to grow Fe-limited cultures at 20 °C and 35 °C (viable range: 22–34 °C). An unsuccessful attempt is defined as cultures exhibiting only negative growth rates and dying within 2–4 days. After all viable cultures (20–34 °C) reached steady-state growth over at least 8 generations, final sampling and data collection were initiated. Although triplicate cultures were grown for each treatment condition, three replicates were excluded from data analysis due to sample losses. These included one P-limited growth rate replicate at 26 °C (final *n* = 2), one P-limited N_2_-fixation rate replicate at 28 °C (final *n* = 2), and one replete iron quota replicate at 20 °C (with the corresponding replete C-IUE and replete N-IUE values; final *n* = 2).

Cultures were grown in temperature-controlled incubators at 150 μmol photons m^−2^ s^−1^ on a 12:12 light:dark cycle. They were maintained in 250 mL polycarbonate bottles using a microwave-sterilized, 0.2 μm-filtered artificial seawater medium amended with the modified Aquil recipe (Sunda et al., 2005), but without added nitrate (NO_3_) (Qu et al., 2022). Semi-continuous dilution of cultures was performed every 2–3 days for at least 4 months to maintain steady-state exponential growth, with the medium amended according to experimental nutrient conditions.

All media was 25 μM EDTA-buffered and received equal concentrations of vitamins [2.97 × 10^−7^ M thiamine (vit. B1), 2.25 × 10^−9^ M biotin (vit. B7), 3.70 × 10^−10^ M cyanocobalamin (vit. B12)] and Aquil trace metals stock (1.21 × 10^−7^ M Mn, 7.97 × 10^−8^ M Zn, 1.00 × 10^−7^ M Mo, and 5.03 × 10^−8^ M Co) (Sunda et al., 2005). Replete and P-limited media were amended with a non-limiting concentration of 250 nM iron (Fe), while Fe-limited cultures had Fe directly added after periodic dilutions to growth-limiting levels of 5 nM. Similarly, with respect to P, replete and Fe-limited media were amended to ample 10 μM Aquil concentrations of P, while P was directly added to P-limited cultures after dilution to limiting concentrations of 0.3 μM. Stringent trace metal clean cultivation protocols were used to ensure Fe limitation (Jiang et al., 2018; Yang et al., 2021). To eliminate contaminating Fe, phosphate stock was passed through an activated Chelex 100 resin column (BioRad Laboratories, Hercules, CA, United States). All amending of nutrients, vitamins, and trace metals were 0.2 μm filter-sterilized and performed with sterilized pipette tips rinsed once with 1% HCL and three times with microwave-sterilized Milli-Q water. Prior to use, all bottles utilized were sequentially immersed for 24 h in 1% Citranox detergent, rinsed with Milli-Q, immersed for a week in 10% HCL, rinsed with Milli-Q, and microwave-sterilized.

Fe and P concentrations used in the study are higher than those typically found in the open ocean, as in most laboratory experiments, given the much higher biomass of experimental cultures relative to natural cell abundances. Higher cell densities are needed in order to maintain adequate biomass for sample analyses. Therefore, limiting concentrations were defined not by absolute nutrient concentrations found in oligotrophic regions, but rather by physiological responses of the cells (concentrations that significantly limited growth rates relative to replete cultures).

Periodic dilutions were based on *in vivo* fluorescence readings with a 10 AU Fluorometer (Turner Designs, San Jose, CA, United States), which were compared and verified with cell counts of 1-mL culture samples (preserved with 0.5% 0.2-μm filtered glutaraldehyde) under an Olympus BX51 fluorescence microscope. The specific growth rate was estimated by the equation μ = (ln D_1_ – ln D_0_)/t, where D refers to cell densities and t represents time in days.

### Elemental stoichiometry

Particulate organic carbon (POC) and nitrogen (PON) concentrations were estimated for each culture across all treatment conditions. Cultures were filtered onto precombusted glass microfiber filters (Whatman, Grade GF/F), oven-dried at ~60 °C, pelleted, and measured with a methionine and acetanilide-calibrated 4,010 Costech Elemental Analyzer (Jiang et al., 2018). Particulate Organic Phosphorus (POP) concentrations were estimated colorimetrically by spectrophotometry (Shimadzu UV-1800) at wavelength 885 nm using samples prepared and analyzed as described in Fu et al. (2005). POC, PON, and POP were normalized to cell counts to determine per-cell elemental quotas.

### N_2_-fixation rates

N_2_-fixation rates were measured using the acetylene reduction assay (Garcia et al., 2013). Duplicate 30 mL samples collected from triplicate cultures were placed into 80 mL sealed-top bottles, injected with 2 mL acetylene into the 50 mL headspace, and left overnight (~12 h). A GC-8a gas chromatograph (Shimadzu Scientific Instruments, Columbia, MD, United States) was used to measure post-incubation ethylene production (reduced acetylene) which was converted to fixed N_2_ using a 3:1 ratio (Fu et al., 2014; Montoya et al., 1996). N_2_-fixation rates were normalized to cell count.

### CO_2_-fixation rates

To estimate CO_2_-fixation rates (net primary productivity), 30 mL subcultures from each replicate were incubated with 0.5 μCi NaH^14^CO_3_ for 24 h (Yang et al., 2021). All were incubated in the same conditions as their experimental treatment (temperature, lighting, etc.), with incubation beginning 2 h after the start of the lighting period. Samples were then filtered on glass microfiber filters (GF/F), stored in darkness overnight, and analyzed using a Beckman LS6000 liquid scintillation counter. Calculated CO_2_-fixation rates were normalized to cell counts to determine CO_2_-fixation.

### Intracellular Fe content and Fe quotas

Culture sampling and measurements of intracellular Fe content were performed as described in Yang et al. (2021). Briefly, 100 mL of samples of replete and Fe-limited cultures were filtered onto acid-washed 0.2 μm Supor polyethersulfone filters (Pall Laboratory), rinsed with oxalate reagent to remove surface-scavenged Fe (Tovar-Sanchez et al., 2003), and digested with 5 mL of indium-amended nitric acid (HNO_3_). The filters were then removed and digested samples were dried overnight, resolubilized in 200 μL equal parts HNO_3_ and hydrochloric acid (HCl), sealed, heated, and left to cool before finally being resuspended in 5 mL of 0.1 M HNO_3_. Inductively coupled plasma mass spectrometry (ICP-MS, Element 2, Thermo Fisher Scientific) was used to analyze the calibrated (0.1–300 ppb metal reference curve) ^56^Fe intensities, which were blank-corrected by subtraction of values measured from blank filters having undergone identical treatment. Fe quotas (mol intracellular Fe per μmol POC) were calculated using measured cellular Fe and cell counts (above).

### Nutrient use efficiencies

Phosphorus Use Efficiency (PUE) is defined as the mol N or C fixed per unit time per unit cellular P (N-PUE, C-PUE, mol N or C fixed h^−1^ mol cellular P^−1^). Similarly, Iron Use Efficiency (IUE) refers to the mol N or C fixed per unit time per unit cellular Fe (N-IUE, C-IUE) (Kustka et al., 2003; Hutchins and Tagliabue, 2024).

### Statistical analysis

RStudio 2021.09.2, JMP Student Edition 18.2.2, and Microsoft Excel 16.86 were used for all statistical analysis and data modeling. Although Brown-Forsythe tests indicated homogeneity of variances across all data sets (*p* > 0.05), some treatment conditions exhibited mild deviations from normality based on the Shapiro–Wilk test (*p* < 0.05). Given the balanced sample sizes, the homoscedasticity of the data, and the well-established robustness of ANOVA to moderate non-normality, the F test was employed to assess differences among groups (Blanca et al., 2017). One-way ANOVA with Tukey’s *post hoc* analysis (*α* = 0.05) was used to determine significance for all parameters relating to *C. watsonii*’s responses to the different treatment conditions. Non-parametric analyses using Kruskal-Wallis tests confirmed significant omnibus effects of temperature on study parameters (N_2_-fixation, CO_2_-fixation, and growth rates) across all treatment conditions (*p* < 0.05).

The measured growth rates, CO_2_-fixation rates, and N_2_-fixation rates under the constant temperatures tested in this experiment were fitted to the thermal response models described by Norberg (2004) and Thomas et al. (2012) to characterize physiological responses to temperature variation. Eppley-Norberg curves were fitted using RStudio 2021.09.2 to estimate various growth temperature parameters, such as optimal growth temperature (T-opt), thermal niche width for growth (based on measured values for T_max_ and T_min_, the maximum and minimum growth temperatures, respectively), maximum CO_2_-fixation rate, and maximum N_2_-fixation rate. T-opt represents the estimated temperature at which growth, CO_2_-fixation, or N_2_-fixation are maximal. Calculated thermal niche width refers to the estimated range of temperatures over which *C. watsonii* demonstrates positive growth, CO_2_-fixation, or N_2_-fixation. Model parameters, with corresponding *R*^2^-values, are reported in Tables 1–3.

**Table 1:** Thermal response curve data for *Crocosphaera watsonii* grown under one of three treatment conditions at 2 °C increments from 20 to 34 °C.

Treatment condition	Maximum growth rate (day^−1^)	Calculated optimum temperature (°C)	Experimental optimum temperature (°C)	Calculated thermal niche width (°C)	Experimental thermal niche width (°C)	Correlation factor (R^2^)	Minimum survival temperature (°C)	Maximum survival temperature (°C)
Replete	0.45	28.73	28–32	15.95	20–34 (14)	0.96	20	34^a^
Iron limited	0.22	29.43	28–32	14.96	22–34 (12)	0.96	22	34^a^
Phosphorus limited	0.21	29.91	28–30	17.54	20–32 (12)	0.95	20	32

Maximum growth rates, calculated optimum temperatures, and calculated thermal niche widths were derived using Eppley–Norberg curves. Experimental optimum temperatures were determined using one-way ANOVA tests (*p* < 0.05). The experimental thermal niche width refers to the range of hospitable temperatures observed for *C. watsonii* during the thermal and nutrient response experiments. Similarly, maximum and minimum survival temperatures refer to the highest and lowest temperatures, respectively, at which growth was observed during the thermal and nutrient response experiments. ^a^Replete and iron-limited cultures survived 2 and a half weeks with measurable growth, carbon-fixation, and nitrogen-fixation.

**Table 2:** Carbon fixation rates for *Crocosphaera watsonii* grown under one of three treatment conditions at 2 °C increments from 20 to 34 °C.

Treatment condition	Maximum carbon fixation rate (pmol C/cell/h)	Calculated optimum temperature for carbon fixation (°C)	Experimental optimum temperature for carbon fixation (°C)	Calculated thermal niche width (°C)	Experimental thermal niche width (°C)	Correlation factor (R^2^)	Minimum carbon fixation temperature (°C)	Maximum carbon fixation temperature (°C)
Replete	0.025	28.69	28–30	17.72	20–34 (14)	0.96	20	34^a^
Iron limited	0.015	30.16	26–34	15.65	22–34 (12)	0.79	22	34^a^
Phosphorus limited	0.015	28.23	28	17.33	20–32 (12)	0.90	20	32

Maximum fixation rates, calculated optimum temperatures, and calculated thermal niche widths for carbon fixation were derived using Eppley–Norberg curves. Experimental optimum temperatures were determined using one-way ANOVA tests (*p* < 0.05). The experimental thermal niche width refers to the range of hospitable temperatures observed for *C. watsonii* during the carbon fixation experiments. Similarly, maximum and minimum survival temperatures refer to the highest and lowest temperatures, respectively, at which carbon fixation was observed. ^a^Replete and iron-limited cultures survived 2 and a half weeks with measurable growth, carbon-fixation, and nitrogen-fixation.

**Table 3:** Nitrogen fixation rates for *Crocosphaera watsonii* grown under one of three treatment conditions at 2 °C increments from 20 to 34 °C.

Treatment condition	Maximum nitrogen fixation rate (pmol N/cell/ h)	Calculated optimum temperature for nitrogen fixation (°C)	Experimental optimum temperature for nitrogen fixation (°C)	Calculated thermal niche width (°C)	Experimental thermal niche width (°C)	Correlation factor (R^2^)	Minimum nitrogen fixation temperature (°C)	Maximum nitrogen fixation temperature (°C)
Replete	0.0024	26.72	26–30	17.80	20–34 (14)	0.87	20	34^a^
Iron limited	0.00062	29.15	28	17.75	22–34 (12)	0.82	22	34^a^
Phosphorus limited	0.00060	27.26	26–28	17.05	20–32 (12)	0.60	20	32

Maximum fixation rates, calculated optimum temperatures, and calculated thermal niche widths for nitrogen fixation were derived using Eppley–Norberg curves. Experimental optimum temperatures were determined using one-way ANOVA tests (*p* < 0.05). The experimental thermal niche width refers to the range of hospitable temperatures observed for *C. watsonii* during the nitrogen fixation experiments. Similarly, maximum and minimum survival temperatures refer to the highest and lowest temperatures, respectively, at which nitrogen fixation was observed. ^a^Replete and iron-limited cultures survived 2 and a half weeks with measurable growth, carbon-fixation, and nitrogen-fixation.

## Results

### Growth rates

**Figure 1:**
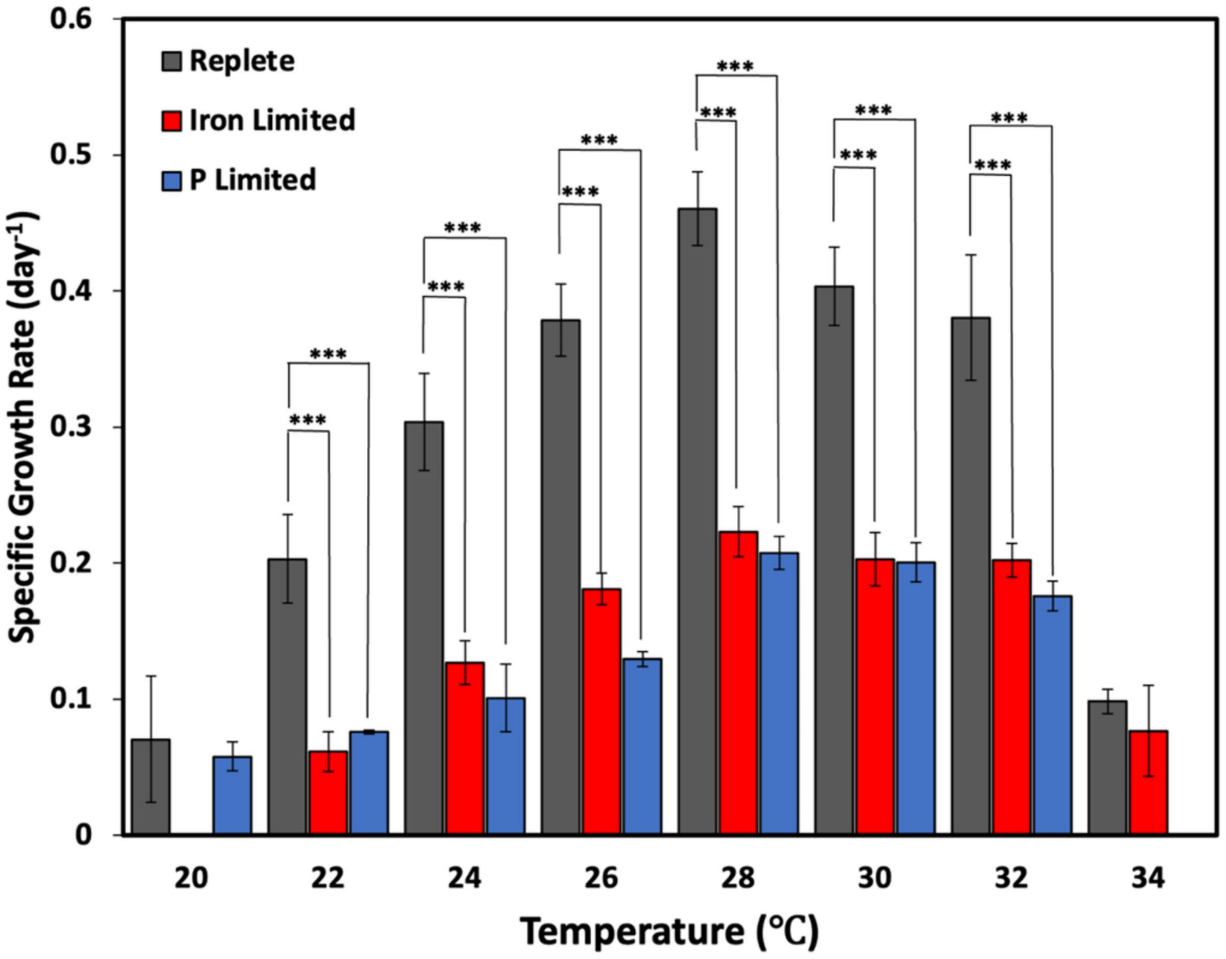
Relative growth rates of *Crocosphaera watsonii* under replete, iron-limited, and phosphorus-limited treatment conditions at eight temperatures (20, 22, 24, 26, 28, 30, 32, and 34 °C). Error bars indicate standard deviation among triplicate cultures at a single temperature and treatment condition. Asterisk brackets indicate the statistical significance of a culture at a specific treatment and temperature relative to replete (***p < 0.001, **p < 0.01, and *p <0.05). Significance tables for differences in growth between temperatures under a single nutrient condition can be found in the supplemental section.

*C. watsonii* cultures grown under Fe- and P-limited nutrient conditions exhibited significantly depressed growth rates relative to replete cultures at all temperatures between 22 and 32 °C (*p* < 0.001) (Figure 1). Thermal modeling derived using Eppley–Norberg curves revealed that the Fe- and P-limited cultures had maximal growth rates of 0.22 day^−1^ and 0.21 day^−1^, respectively, representing a 51 and 53% decreases relative to the 0.45 day^−1^ growth rate seen under replete conditions (Table 1). A one-way ANOVA test revealed that the growth rates of *C. watsonii* did not vary significantly at 28, 30, and 32 °C under replete and Fe-limited conditions, indicating that Fe-limitation does not seem to alter the optimum temperature niche of *C. watsonii* (Supplementary Tables S1, S2; Figure 1). However, under P-limited conditions, a one-way ANOVA test revealed that the optimum temperature niche of *C. watsonii* was narrower, between 28 and 30 °C (Supplementary Table S3; Figure 1).

**Figure 2:**
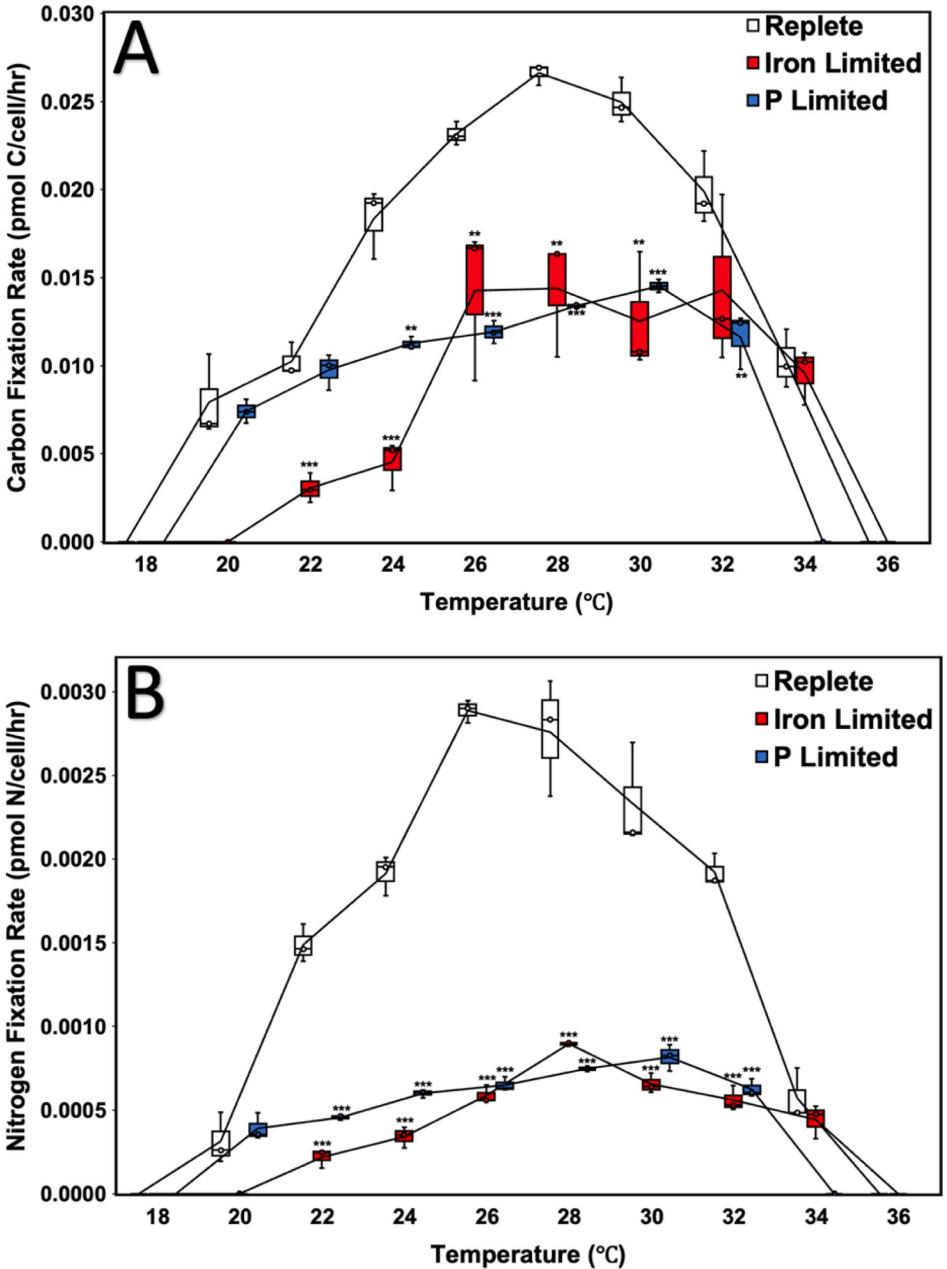
Carbon fixation **(A)** and nitrogen fixation **(B)** rates of *Crocosphaera watsonii* under replete, iron-limited, and phosphorus (P)-limited treatment conditions at eight temperatures (20, 22, 24, 26, 28, 30, 32, and 34 °C). Error bars indicate standard deviation among triplicate cultures at a single temperature and treatment condition. Asterisks indicate the statistical significance of a culture at a specific treatment and temperature relative to replete (***p < 0.001, **p < 0.01, and *p <0.05). For example, in **(A)**, at 22 °C, the carbon fixation rates of the iron-limited cultures were significantly lower than replete (*p* < 0.001), whereas the carbon fixation rates of phosphorus (P)-limited cultures were not. Significance tables for differences in carbon and nitrogen fixation rates between temperatures under a single nutrient condition can be found in the supplemental section.

At the coldest point of the thermal response curve, 20 °C acclimated replete and P-limited cultures exhibited significantly reduced but measurable growth rates of 0.070 and 0.058 day^−1^, respectively, with no statistical difference in their growth (Figure 1). Conversely, Fe-limited cultures failed to grow at 20 °C after three unsuccessful attempts (Figure 1). At the warmest point of the thermal response curve, P-limited cultures were unable to grow at 34 °C, with three unsuccessful attempts. In contrast, replete and Fe-limited cultures grew and carried out CO_2_ and N_2_ fixation at 34 °C for 17 days, exhibiting growth rates of 0.099 and 0.077 day^−1^, respectively, which were not statistically different (Tables 1–3; Figures 1, 2). Afterwards, however, growth and fixation rates began to decline, and cultures could no longer sustain growth at such a high temperature.

### CO_2_-fixation and N_2_-fixation rates

Relative to replete cultures, Fe-limited cultures fixed CO_2_ at a significantly reduced rate at all tested temperatures between 22 and 30 °C (*p* < 0.01) (Figure 2A). Similarly, P-limited cultures exhibited significantly reduced CO_2_-fixation at temperatures between 24 °C and 32 °C (*p* < 0.01) (Figure 2A). However, Fe- and P-limited cultures did not exhibit significant differences in CO_2_-fixation rates relative to replete cultures between 32 and 34 °C and 20–22 °C, respectively (Figure 2A). Overall, replete cultures exhibited a distinct unimodal curve, with CO_2_-fixation rates peaking at 0.027 pmol C/cell/h at 28 °C (Figure 2A). In contrast, the thermal response curves of cultures under nutrient limitation were significantly flattened (Figure 2A).

At the minimum point of the thermal response curve (20 °C), P-limited cultures fixed C at a rate of 0.0074 pmol C/cell/h, representing only a 48.8% decrease relative to the 0.015 pmol C/cell/h rate observed at 30 °C (*p* < 0.001) (Figure 2A). On the other hand, P-limited cultures at the maximum point of the thermal response curve (32 °C) fixed C at a rate of 0.012 pmol C/cell/h, a 19.9% decrease relative to the rate observed at 30 °C (Figure 2A).

As for the Fe-limited cultures grown at the colder end of the thermal response curve (22 °C), significantly reduced rates of CO_2_-fixation were measured relative to replete cultures (*p* < 0.001) (Figure 2A). In contrast, at 20 and 22 °C, the CO_2_-fixation rates of the P-limited cultures were comparable to their replete counterparts (Figure 2A). At the warmer end of the thermal response curve, the CO_2_-fixation rates of the Fe-limited cultures were not significantly different from those of replete cultures at 32 and 34 °C (Figure 2A). In contrast, the P-limited cultures at 32 °C fixed C at a significantly reduced rate as compared to the replete cultures (*p* < 0.01) (Figure 2A). Compared to their growth rates, which plateaued between 28 and 32 °C, the plateau for the CO_2_-fixation rates of the Fe-limited cultures was significantly wider, between 26 and 34 °C (Figures 1, 2A; Supplementary Table S5). On the other hand, P-limited cultures exhibited a distinct peak in their CO_2_-fixation rates at 30 °C, while their growth rates plateaued between 28 and 30 °C (Figures 1, 2A; Supplementary Tables S3, S6).

With regards to the N_2_-fixation rates of *C. watsonii,* replete cultures again exhibited a distinct unimodal curve, with N_2_-fixation peaking at 0.0029 pmol N/cell/ h at 26 °C (Figure 2B). Further, N_2_-fixation in the P-limited cultures peaked at 30 °C at a rate of 0.0008 pmol N/cell/h. In comparison, the N_2_-fixation rates of the Fe-limited cultures peaked at 28 °C at a rate of 0.0009 pmol N/cell/ h (Figure 2B). This contrasted with the CO_2_-fixation rates of the Fe-limited cultures, which exhibited a much broader peak between 26 and 34 °C (Figure 2A). As compared to their growth rates, which plateaued between 28 °C and 32 °C, the N_2_-fixation rates of the Fe-limited cultures had a much narrower peak at 28 °C (Figures 1, 2B; Supplementary Tables S2, S8). Additionally, the growth rates of the P-limited cultures plateaued between 28 and 30 °C, while their N_2_-fixation rates plateaued between 26 and 32 °C (Figures 1, 2B; Supplementary Tables S2, S9).

Overall, the N_2_-fixation rates of *C. watsonii* appeared to be significantly more impacted by nutrient limitation than the CO_2_-fixation rates (Figures 2A,B; Tables 2, 3). The most significant decreases in the CO_2_-fixation rates of the Fe- and P-limited cultures relative to replete cultures were 75.2% (24 °C) and 49.7% (28 °C), respectively (Figure 2A). In comparison, the N_2_-fixation rates of the Fe- and P-limited cultures were reduced by as much as 85.3% (22 °C) and 77.4% (26 °C), respectively, relative to replete cultures (Figure 2B). The average percent declines in CO_2_-fixation across all temperatures of the Fe- and P-limited cultures relative to replete cultures were 44.9 and 33.1%, respectively. In contrast, the average percent declines in N_2_-fixation of the Fe- and P-limited cultures relative to replete cultures were 68.6 and 56.5%, respectively (Figures 2A,B).

### Iron and phosphorus quotas

**Figure 3:**
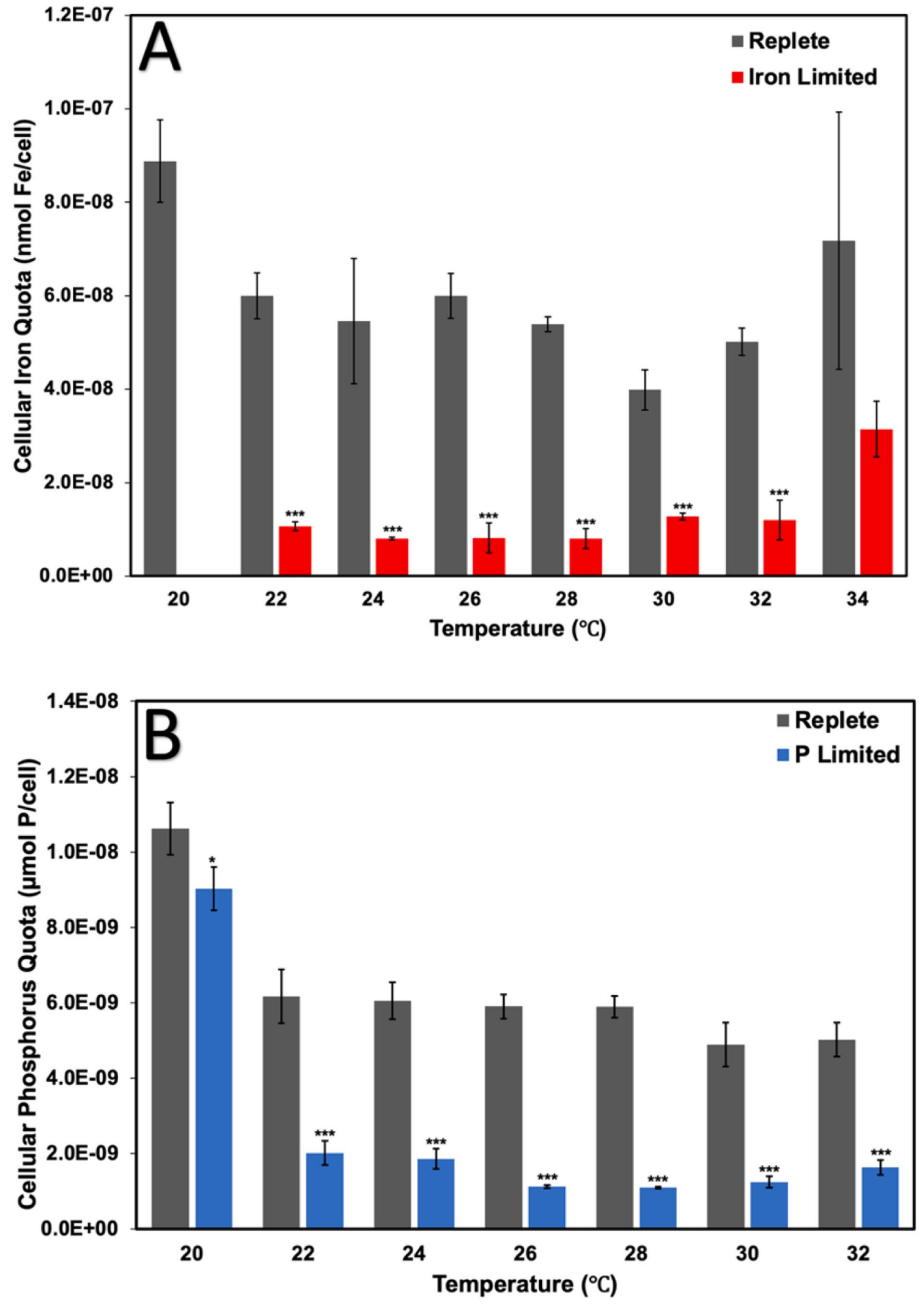
Cellular iron quotas of replete and iron-limited cultures **(A)** and cellular phosphorus quotas of replete and phosphorus-limited cultures **(B)** of *Crocosphaera watsonii* at eight temperatures (20, 22, 24, 26, 28, 30, 32, and 34 °C). Error bars indicate standard deviation among triplicate cultures at a single temperature and treatment condition. Asterisks indicate the statistical significance of a culture at a specific treatment and temperature relative to replete (***p < 0.001, **p < 0.01, and *p <0.05).

Unsurprisingly, Fe-limited cultures were found to possess significantly diminished cellular Fe quotas relative to replete cultures at all temperatures between 22 °C and 32 °C (*p* < 0.001) (Figure 3A). At the colder end of the thermal response curve, 22 °C acclimated Fe-limited cultures contained 82% less Fe per cell relative to replete cultures (*p* < 0.001) (Figure 3A). Conversely, at the warmer end of the thermal response curve, 34 °C acclimated Fe-limited cultures contained 51% less Fe per cell relative to replete cultures, though the difference was not statistically significant (Figure 3A). The most notable disparities in cellular Fe quotas were found at 26 and 28 °C at 86 and 85% less than replete cultures, respectively (*p* < 0.001) (Figure 3A).

Similarly, P-limited cultures incubated at temperatures between 22 and 32 °C saw significant decreases in cellular P quotas relative to replete cultures (*p* < 0.001) (Figure 3B). Although statistically significant, the 20 °C acclimated P-limited cultures at the colder end of the response curve contained only 15% less P per cell relative to replete cultures (*p* < 0.05) (Figure 3B). In contrast, the 32 °C acclimated P-limited cultures at the warmer end of the thermal response curve contained 67% less P per cell relative to replete cultures (*p* <0.001) (Figure 3B). The largest differences in cellular P quotas were found at 26 and 28 °C at 81 and 81% less than replete cultures, respectively (*p* < 0.001) (Figure 3B).

### Iron and phosphorus use efficiencies

**Figure 4:**
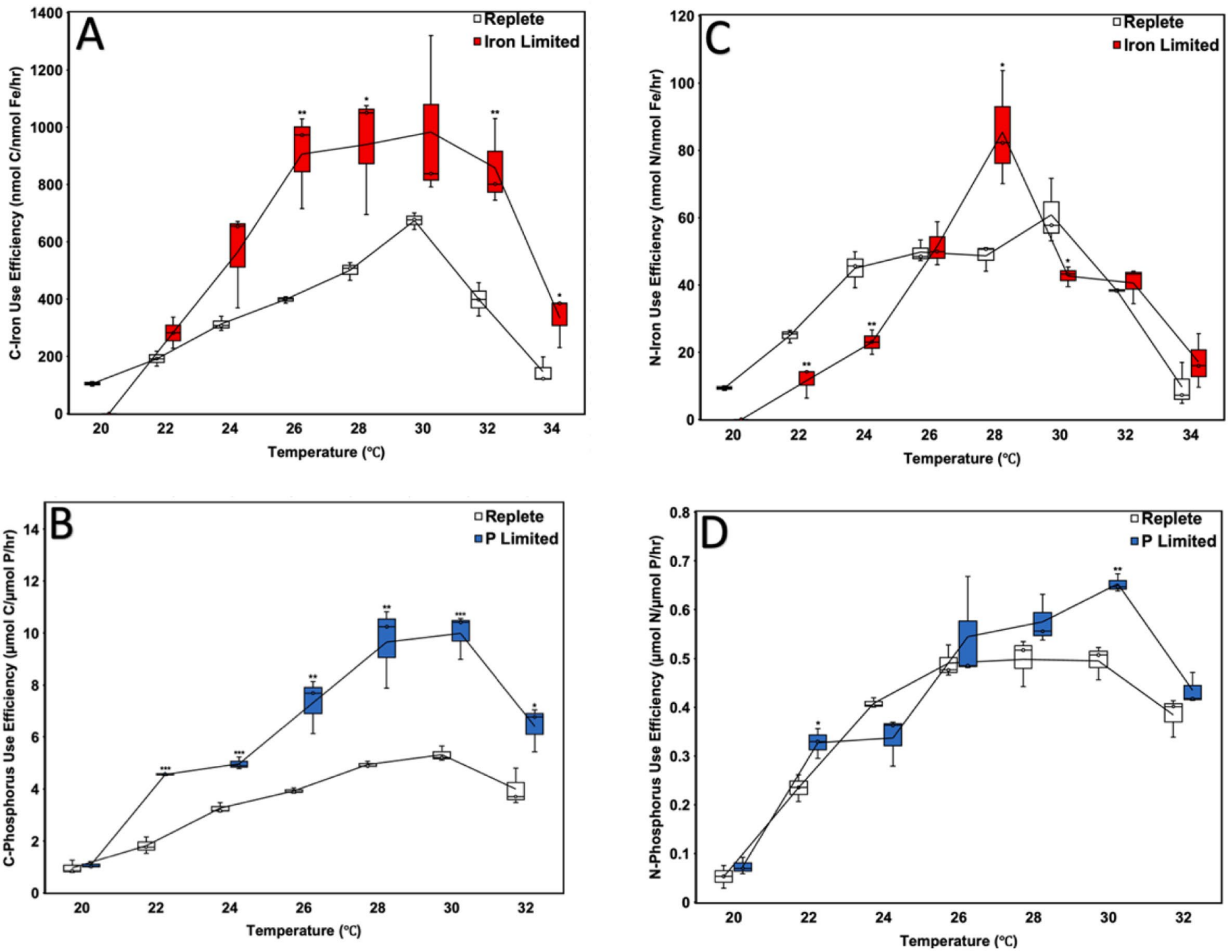
Carbon-specific iron **(A)** and phosphorus **(B)** use efficiencies as well as nitrogen-specific iron **(C)** and phosphorus **(D)** use efficiencies of *Crocosphaera watsonii* cultures at eight temperatures (20, 22, 24, 26, 28, 30, 32, and 34 °C). Error bars indicate standard deviation among triplicate cultures at a single temperature and treatment condition. Asterisks indicate the statistical significance of a culture at a specific treatment and temperature relative to replete (***p < 0.001, **p < 0.01, and *p <0.05).

Carbon-specific Iron Use Efficiency (C-IUE) generally increased with nutrient limitation, with significantly greater C-IUE observed in Fe-limited cultures incubated at 24, 26, 28, 32, and 34 °C relative to replete cultures (*p* < 0.05) (Figure 4A). Further, C-IUE was notably greater than replete cultures at 30 °C, though the difference was not statistically significant (Figure 4A). However, there was no significant difference in the C-IUE of the Fe-limited cultures relative to replete cultures at their minimum survival temperature of 22 °C (Figure 4A).

Similarly, Carbon-specific Phosphorus Use Efficiency (C-PUE) was also observed to increase under nutrient limitation, with significantly greater C-PUE observed in all P-limited cultures incubated at temperatures between 22 and 32 °C relative to replete cultures (*p* < 0.05) (Figure 4B). However, there was no statistically significant difference in the C-PUE of the P-limited cultures incubated at their minimum survival temperature of 20 °C relative to replete cultures (Figure 4B).

Unlike C-IUE and C-PUE, however, the Nitrogen-specific Fe and P Use Efficiencies (N-IUE and N-PUE) did not exhibit clear trends in nutrient use efficiency (Figures 4C,D). While the Fe-limited cultures at 22, 24, and 30 °C exhibited statistically significant reductions in N-IUE relative to replete cultures, Fe-limited cultures at 28 °C saw increased N-IUE (*p*-value<0.05; Figure 4C). Conversely, Fe-limited cultures at 28, 32, and 34 °C did not exhibit statistically significant changes in N-IUE (Figure 4C). Likewise, P-limited cultures at 22 and 30 °C exhibited significant increases in N-PUE relative to replete cultures (*p* < 0.05) (Figure 4D), while the N-PUE of P-limited cultures at all other temperatures tested did not differ significantly from replete cultures.

## Discussion

### Interactive temperature and nutrient limitation effects on growth and fixation rates

While the growth and fixation rates of the replete cultures tended toward narrower unimodal-like curves with especially decreased performance toward the ends of the temperature spectrum, the nutrient-limited cultures demonstrated a greater degree of plateauing; differences across temperatures were relatively less under limiting conditions (Figures 1, 2). A decrease in temperature dependency of metabolic rates during nutrient limitation has also been similarly observed in phytoplankton under nitrogen-limited conditions (Marañón et al., 2018). Despite this similar trend found under both Fe-limited and P-limited conditions, each showed disparate responses at opposite ends of the temperature spectrum. Fe-limited cultures tended to perform better at higher temperatures and showed the same thermal optimum plateau as replete cultures for growth rates (28–32 °C), while P-limited cultures performed better at lower temperatures, demonstrating a narrower, decreased optimum range (28–30 °C) (Table 1). The similarities and differences observed across both limitations may be partially explained by previous findings that *C. watsonii* demonstrates both shared and distinct molecular-mechanism responses to Fe-limited and P-limited conditions (Yang et al., 2022). Transcriptome analyses have revealed shared patterns of upregulation of certain diel genes across both conditions, as well as unique processes such as more rapid protein turnover under Fe-limitation, and environmental sense-and-respond systems that were upregulated under P-limited conditions (Yang et al., 2022).

Another finding of the study suggests that nutrient limitation more negatively impacts N_2_-fixation rates than CO_2_-fixation rates (Figure 2). We hypothesize that differences in the resource usage efficiencies observed may have resulted from this differential impact, as both C-IUEs and C-PUEs were typically significantly higher for the nutrient-limited cultures than the replete cultures, while N-IUEs and N-PUEs were typically lower or at replete levels (Figure 4). While similar findings have been found with other limitation studies regarding greater impacts on N- than CO_2_-fixation, use efficiency data is varied, and studies were attempted at only one or three temperature conditions (Jacq et al., 2014; Yang et al., 2021, 2022). Given our results, nutrient limitation (rather than temperature) may be serving as the proximate growth-limiting factor, since N_2_-fixation rates were disproportionately impacted regardless of temperature condition (except at the extremes where temperature inhibition may have offset nutrient limitation effects) (Figure 2). Since *C. watsonii* partitions its CO_2_-fixation to the daytime and N_2_-fixation to the night, it is possible that changes to this diel cycle in response to limitation (as mentioned above in the study of molecular mechanisms) create more disruption to the N_2_-fixation cycle than the CO_2_-fixation cycle (Masuda et al., 2022; Yang et al., 2022).

### Temperature effects dominate over Fe-limitation at higher temperatures and P-limitation at lower temperatures

Our study supports former findings that increasing temperatures may decrease the negative effects of Fe-limitation on the growth and fixation rates of *C. watsonii*, positioning *C. watsonii* to be successfully selected for in future warmer, Fe-limited conditions (Yang et al., 2021). While Yang et al. (2021) similarly observed increases in *C. watsonii* growth and function between 22 and 27 °C, a direct comparison of viable replete and Fe-limited cultures at its upper thermal limit was not performed. Our results at the experimental temperature max (34 °C) showed that differences in growth and fixation rates between Fe-limited and replete conditions became insignificant (Figure 1). This suggests that thermal stress at sufficiently high temperatures may outweigh and override Fe-limitation physiological impacts, an effect that was not detectable within a narrower temperature range.

Although this Fe-warming effect is still being investigated, previous studies have shown that the rates of many Fe-enriched metabolic pathways (photosynthesis, CO_2_-fixation, respiration, and enzyme catalysis) to potentially be thermally sensitive in phytoplankton (Hutchins and Tagliabue, 2024). Another theory suggests that decreased cell size associated with increased Fe-limitation (Jacq et al., 2014) and increased temperature (Finkel et al., 2009; Polovina and Woodworth, 2012; Yang et al., 2021) may help to improve survivability in limiting conditions. This is because smaller cell sizes increase the surface area to volume ratio, leading to more efficient uptake, while at the same time decreasing cellular Fe quotas and thus Fe requirements. Finally, a recent study on diatoms spanning various Fe concentrations and three temperatures found a decreased Fe half-saturation coefficient (K_Fe_) for growth at higher temperatures, indicating that in warming conditions, less Fe may be required to maintain the same growth rates (Jabre and Bertrand, 2020). *C. watsonii’s* K_Fe_ responses to temperature changes remain to be investigated. However, previous studies have found *C. watsonii’s* K_Fe_ to be half the magnitude of other prominent diazotrophs, such as *Trichodesmium*, suggesting it is less impacted by Fe-limitation and better suited to thrive under increasingly Fe-limited conditions (Jacq et al., 2014).

P-limited cultures showed relatively opposite results to Fe-limited cultures, with temperature’s effects offsetting P-limitation’s impacts most strongly at the cooler end; at 20 °C, there was no significant difference in growth and fixation rates between P-limited and replete conditions (Figure 1). Deng et al.’s study on P-limited *C. watsonii* did not explore temperatures cooler than 25 °C and determined that high temperatures (i.e., 31 °C) could potentially reduce the effects of P-limitation in *C. watsonii* (2021). The trend of our P-limited cultures at higher temperatures (at 30, 32 °C, and inability to grow at 34 °C) compared to replete and Fe-limited cultures suggests that higher temperatures may exacerbate P-limitation effects at the extreme ends, and P-limitation may make cultures less thermally-resilient in warmer waters. Our studies may vary because (a) our results explore the full curve from 20 to 32 °C (not just at 25, 28, and 31 °C), (b) our cultures were more nutrient-limited in terms of growth rates (P-limited growth rates in our study were half those of replete rates, while Deng et al. P-limited growth rates were closer to replete rates at those three temperatures), and (c) we used a different strain. Similar to Fe, former research has also suggested the rates of some P-enriched metabolic pathways [protein synthesis (ribosomes, rRNA) and energy metabolism (ATP hydrolysis)] can potentially be thermally sensitive in phytoplankton (Hutchins and Tagliabue, 2024). While previous studies have been conducted on P-limitation and warming for *C. watsonii*, including transcriptional analysis, no research has been conducted at colder temperatures below 25 °C (Deng et al., 2021). However, a study of 5 species of freshwater phytoplankton [*Ankistrodesmus, Chlamydomonas, Monoraphidium, Scenedesmus,* and *Raphidocelis*] under P-limitation has theorized optimal temperature for growth (T-opt) to be a saturating function of nutrient concentrations (specifically, P and nitrate); greater P-limitation was associated with decreases in T-opt for growth rates, ranging from declines in T-opt of 2–15 °C compared to their replete counterparts, depending on the species (Bestion et al., 2018a; Thomas et al., 2017). The half-saturation constant of P for growth rates has also been found to increase with temperature for P-limitation for some species, possibly suggesting a larger P requirement at higher temperatures (Bestion et al., 2018b). However, the mechanisms underlying these responses are still poorly investigated.

Furthermore, extreme temperatures suppress growth rates, and so directly translate to longer cell cycles (Zhao et al., 2022). This may allow greater time for the accumulation of nutrients (thus reducing the nutrient limitation effects) as cells are slower or unable to divide. This could account for the increased cellular P quotas we observed at lower temperatures, as well as the similar performance of replete and P-limited cultures observed for P-limitation at 20 °C (with the highest amounts of cellular P) (Figure 3B). Similarly, at the higher temperature extreme, with similarly lower growth rates, we observed the highest intracellular Fe quota potentially due to the same reasoning (Figure 3A). Beyond growth rates, additionally, upregulation of P-scavenging genes at higher temperatures may account for the increased P quota observed at 32 °C (Figure 3B; Dyhrman and Haley, 2006).

### Maximum and minimum temperatures

In Fe-limited conditions, *C. watsonii* had a maximal survival temperature of 34 °C, similar to that of replete cultures. P-limitation, however, decreased the maximal survival temperature to 32 °C, suggesting that survival mechanisms are unable to maintain cell viability at this temperature (Table 1). While the specific molecular drivers for this loss of viability are unknown without proteomic or transcriptomic data, we hypothesize that it may be due to the thermal denaturation of proteins necessary for phosphate uptake (pstS and sphX), intracellular recycling (ppX), and utilization, which have been shown to be upregulated under elevated temperatures and P-limited conditions (Deng et al., 2021). Denaturation of proteins used in any of these pathways may limit phosphate acquisition, reducing available ATP and inhibiting transcription and translation of gene products necessary for survival. While genes coding for these proteins have been shown to be transcribed at 31 °C (Deng et al., 2021), transcriptomes beyond this temperature are necessary to determine if they play a role *in C. watsonii*’s inability to survive at 34 °C.

Conversely, replete and P-limited cultures had a minimum survival temperature of 20 °C, while Fe-limited cultures could not survive below 22 °C (Table 1). One reason may be that a similar process of thermal denaturation of important proteins for Fe intake or other processes may be occurring at the cold end at the thermal curve as well, however no proteome data yet exist for Fe-limitation at lower temperatures for *C. watsonii.* Additionally, it may be due to the potentially lesser Fe requirement at higher temperatures (and consequently a greater requirement at lower temperatures) as mentioned previously (Jabre and Bertrand, 2020).

Another hypothesis for Fe-limited cultures’ inability to survive at the cooler end could be attributed to the combined negative effects of temperature and Fe-limitation on photosynthetic activity. Severe Fe-limitation has been found to decrease photosynthesis rates, as indicated by significantly reduced CO_2_-fixation rates and chlorophyll a cellular concentration in *C. watsonii* (Jacq et al., 2014). Furthermore, while no transcriptomic analyses have yet been conducted on *C. watsonii* at its temperature minimum, transcriptomic analyses of another unicellular cyanobacterium *Prochlorococcus* found downregulation of photosystem I and photosystem II transcripts at colder temperatures during the daytime (Partensky et al., 1999; Masuda et al., 2022; Alonso-Sáez et al., 2023). Furthermore, a study on the freshwater unicellular cyanobacterium *Synechocystis* found that even in cases of upregulation of photosynthetic genes due to colder temperatures, cold stress significantly decreased translational efficiency, especially for photosynthetic reaction center proteins, impacting the overall photosynthetic yield (Cho et al., 2021). Thus, it is possible that a combination of Fe-limitation and cold stress could alter photosynthetic systems to unsustainable levels, shifting the temperature minimum upwards. However, to test whether these cold stress findings also apply to *C. watsonii*, transcriptomic and proteomic analyses at its temperature minimum in Fe-limited and replete conditions are needed.

### The future of *Crocosphaera*: SSP5-8.5 scenario and marine heat waves

Based on a literature review conducted by Moisander et al. (2010), *C. watsonii* is found primarily in tropical and subtropical gyres between 30°N–S in the Pacific and 25°N–S in the Atlantic. Future distributions of this diazotroph and other cyanobacteria (namely, *Trichodesmium*) have been modeled previously using the IPCC’s “business as usual” RCP8.5 emissions scenario along with growth and fixation data (IPCC, 2014; Jiang et al., 2018; Yang et al., 2021). A more recent model integrated thermal curves and nutrient limitation with elemental use efficiencies (EUEs) and other drivers, and it predicted a 15% decline in global N_2_-fixation rates of *C. watsonii* by the century’s end, contrary to previous models (Wrightson et al., 2022). This decrease was largely due to changes in biogeographic distribution due to low-latitude tropical ocean temperatures that exceed thermal limits but was predicted to be greater if partially offsetting thermal effects on EUEs were not taken into account.

While our study does not include modeling projections, we used the IPCC’s WGI Interactive Atlas to examine long-term temperature projections for 2081–2,100 under the updated SSP5-8.5 scenario (which corresponds to the RCP8.5 scenario) (Abram et al., 2019; Gutiérrez et al., 2021). In the northern hemisphere subtropics, sea surface temperatures (SSTs) are expected to increase compared to a 1995–2014 baseline by ~3 °C, with an annual median of 28.1 °C by 2,100, and a median of 31 °C specifically from August–October. Similarly, the predicted increase in the southern hemisphere subtropics is 2.6 °C (median: 25.9 °C annually, 28.5 °C in Feb–Mar). These data suggest that warming oceans will reach *C. watsonii’s* thermal optima range (28–32 °C for replete and Fe-limited cultures, 28–30 °C for P-limited cultures) in the subtropical gyres, positioning *C. watsonii* to thrive and be successful in these regimes in the upcoming century, even in oligotrophic conditions (Table 1). Previous studies have also indicated that *C. watsonii* has some limited potential to acclimate and adapt to selection under high temperatures (Qu et al., 2022).

However, despite this promising outlook, median projections do not consider the growing concerns regarding the impacts of transient marine heat waves, which are expected to increase in frequency, intensity, and duration over this century (Oliver et al., 2019). For example, events like the 2010–2011 Western Australia and 2013–2016 Northeast Pacific heat waves experienced transient SST anomalies of +5 and +3–6.2 °C, respectively (Pearce and Feng, 2013; Gentemann et al., 2017). Therefore, while global median SSTs are not predicted to exceed 33 °C by 2,100, sudden heat waves of +1–6 °C in the tropics and subtropics may have detrimental effects on *C. watsonii*’s survivability (>32 °C for P-limited cultures, >34 °C for Fe-limited and replete cultures) (Gutiérrez et al., 2021). Already, heat events are breaking SST records and surpassing *C. watsonii*’s viable range, such as in July 2023, when a buoy near southern Florida observed a record 38.4 °C SST (NOAA National Data Buoy Center, 2023).

Additionally, a study conducted by Athanase et al. (2024) suggested that the 2019/2020 Northeast Pacific marine heat wave would likely have covered a greater area and been more intense if it had occurred in a climate that was +4 °C warmer. As the SSP5-8.5 scenario predicts a 3.5°C increase in global surface atmospheric temperature by 2100 (compared to a 1984-2014 baseline), it is reasonable to hypothesize that future marine heat waves may be more physically expansive with even greater temperature deviations than the extreme events we are currently observing (Intergovernmental Panel on Climate Change (IPCC), 2021). Therefore, in considering maximal survival temperatures and transient temperature spikes, nutrient limitation (P vs. Fe) may need to be considered when predicting thermal resilience. While oligotrophic ocean biogeochemistry is still being researched, studies have generally found the North Atlantic to be P-limited and the Pacific Ocean to be Fe-limited, though overlap exists (Martiny et al., 2019; Browning and Moore, 2023).

Drawing on predicted sea surface temperatures, oceanic nutrient conditions, and the results of our experiment, we propose the following hypotheses regarding shifts in the global distribution of *C. watsonii,* assuming UCYN-B populations in these regions have similar temperature-nutrient interaction responses to our model strain WH0005. While strain-level diversity in some traits is observed in *C. watsonii* natural populations (Webb et al., 2009), WH0005 may be an appropriate model isolate for this study as it has near-identical replete thermal curves to other strains isolated from the North Pacific, North Atlantic, and South Atlantic (WH0003, WH0402, WH0401) (Fu et al., 2014).

Thus, in a P-limited Atlantic reaching ~31 °C, a 3 °C heat wave could, in principle, make this region uninhabitable for *C. watsonii.* P-limited cultures did not survive at 34 °C and would be unlikely to endure a short-term heating event. Given P-limited cultures’ better performance in lower temperatures and the expected northern movement of isotherms due to global warming, it seems possible that *C. watsonii* in the Atlantic may move poleward into cooler temperature habitats, with the potential to live in latitudes as cool as 20 °C, reaching the 45° north and 31° south parallels. This is in line with a previous modeling study that predicts a doubling of the area of thermal stress experienced by *Crocosphaera* by the end of the century, suggesting a reduced presence in the lower latitudes but expansion into higher latitudes, increasing its range by up to 10% more of the ocean than was previously survivable due to cold temperatures (Wrightson et al., 2022).

On the other hand, in the Pacific Ocean, extrapolation from our experiments suggests Fe-limited *C. watsonii* may be able to survive heat wave events of similar magnitudes. Given that replete and Fe-limited cultures were able to survive for approximately 2 weeks at the maximum temperature of 34 °C, this indicates the potential for *C. watsonii* to survive transient fluctuations in temperature like marine heat waves for a short period of time if Fe-limited. However, models also suggest that *Crocosphaera*’s response in the Pacific is more complicated and variable than in the Atlantic. This results from further factors impacting the Pacific, like projected concentrations of fixed nitrogen, which also play a role in determining the diazotroph niche compared to non-nitrogen-fixing species (Inomura et al., 2019b; Wrightson et al., 2022).

## Conclusion

As a growing body of research continues to reveal the globally important role of *C. watsonii*, our complete temperature curve contributes to a more comprehensive understanding of nutrient-temperature trends. Assuming other strains act similarly to our isolate, as previously mentioned, our study suggests that P-limited *C. watsonii* may have an advantage at lower temperatures and be less thermally resilient at higher temperatures, compared to those growing in Fe-limited regions in which the opposite was observed. Thus, given predicted increases in long-term SSTs and oceanic heat waves, we suggest that *C. watsonii* in the Pacific Ocean (Fe-limited) may be a more resilient survivor under future global warming scenarios of 2,100. In contrast, *C. watsonii* in the North Atlantic (P-limited) may face greater thermal vulnerability and is more likely to move poleward into cooler latitudes, making low latitude regimes potentially more vulnerable to nitrogen limitation.

Considering these outcomes, we suggest future research be conducted on a wide diversity of *C. watsonii* strains regarding temperature and nutrient interactions and how they affect oligotrophic resilience. Although our study tested *C. watsonii*’s different responses to Fe and P limitation individually, we also suggest that co-limitation should be explored, as recent studies suggest a high prevalence of co-limitation globally of various elements (Browning and Moore, 2023). Previous papers examining the effects of Fe and P co-limitation on *C. watsonii* have suggested that Fe limitation can moderate the effects of P limitation, resulting in a phenotype with increased viability that can outperform singularly P-limited cultures (Garcia et al., 2014; Yang et al., 2022). Co-limited cells may also perform more similarly to singular limitation, based on which nutrient is more limiting. While our study did not consider the interaction between co-limitation and temperature, these findings pose a possibility for P-limited *C. watsonii* to be able to survive at 34 °C if co-limited by Fe, potentially allowing their survival during transient oceanic heat waves. However, temperature curves and transcriptomic analysis of co-limited cultures are needed to accurately assess their viability and changes in molecular mechanisms at different temperatures. Future research should attempt temperature curves of multiple *C. watsonii* strains and include co-limitation along with transcriptome or proteome analyses for increased generalizability and mechanistic insights into *C. watsonii’*s future across differing oligotrophic regimes across the ocean. Likewise, because of the different thermal responses of Fe and P limitations, it may be beneficial for future modeling studies to use such curve data as well as to carefully consider the contrasting nutrient limitations of different regimes in their calculations. Finally, fieldwork with populations of *Crocosphaera* and other natural populations in the Atlantic and Pacific during heat wave events would also be very useful in studying their response to high and sudden thermal stress events.

## Data Availability

The raw data supporting the conclusions of this article will be made available by the authors, without undue reservation.
