# Supplementary material for: Phosphorus limitation heightens vulnerability of Crocosphaera watsonii to ocean warming compared with iron limitation
Source: Front Microbiol. 2026 Jan 6;16:1718897. doi: 10.3389/fmicb.2025.1718897 (PMC12815713; doi:10.3389/fmicb.2025.1718897)
Supplement: Supplementary file 1 [file Data_Sheet_1.docx]

**Supplemental**

***Growth Rate Significance Tables***

**Table 4.** Statistical significance table of the growth rates of replete cultures at various temperatures.

**
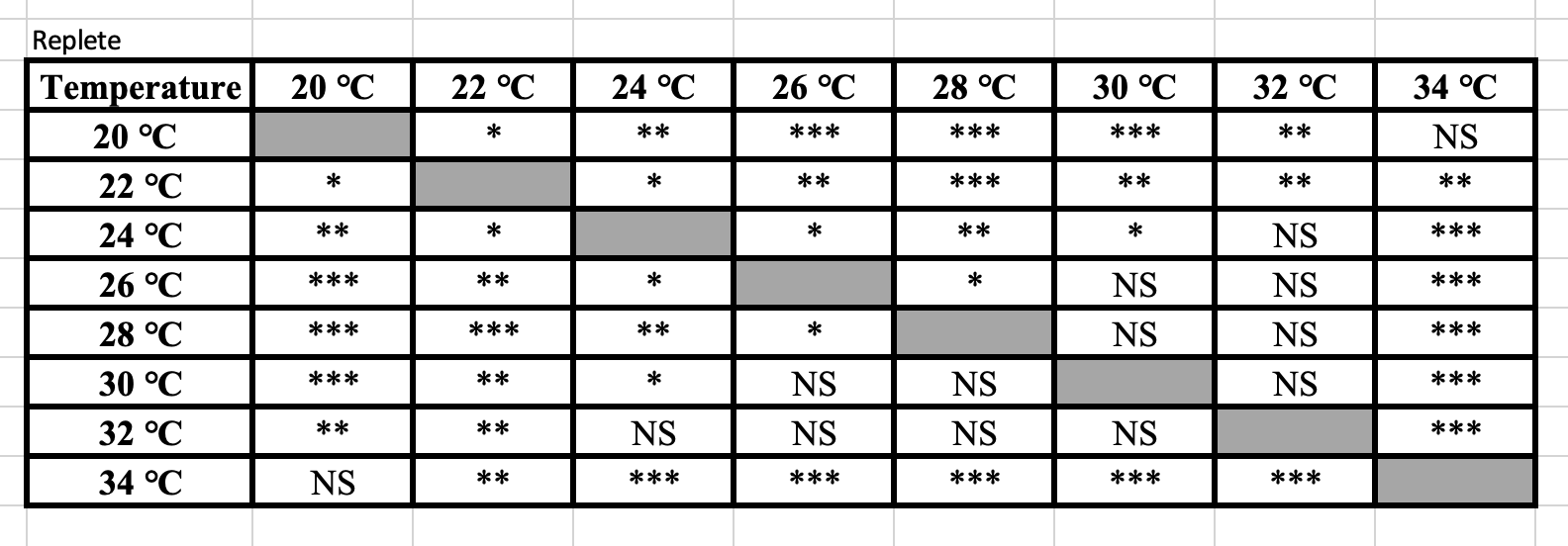
**

Asterisks indicate significance between temperatures: *** = *p* < 0.001, ** = *p* < 0.01, * = *p* < 0.05, and NS = not significant.

**Table 5.** Statistical significance table of the growth rates of iron limited cultures at various temperatures.

**
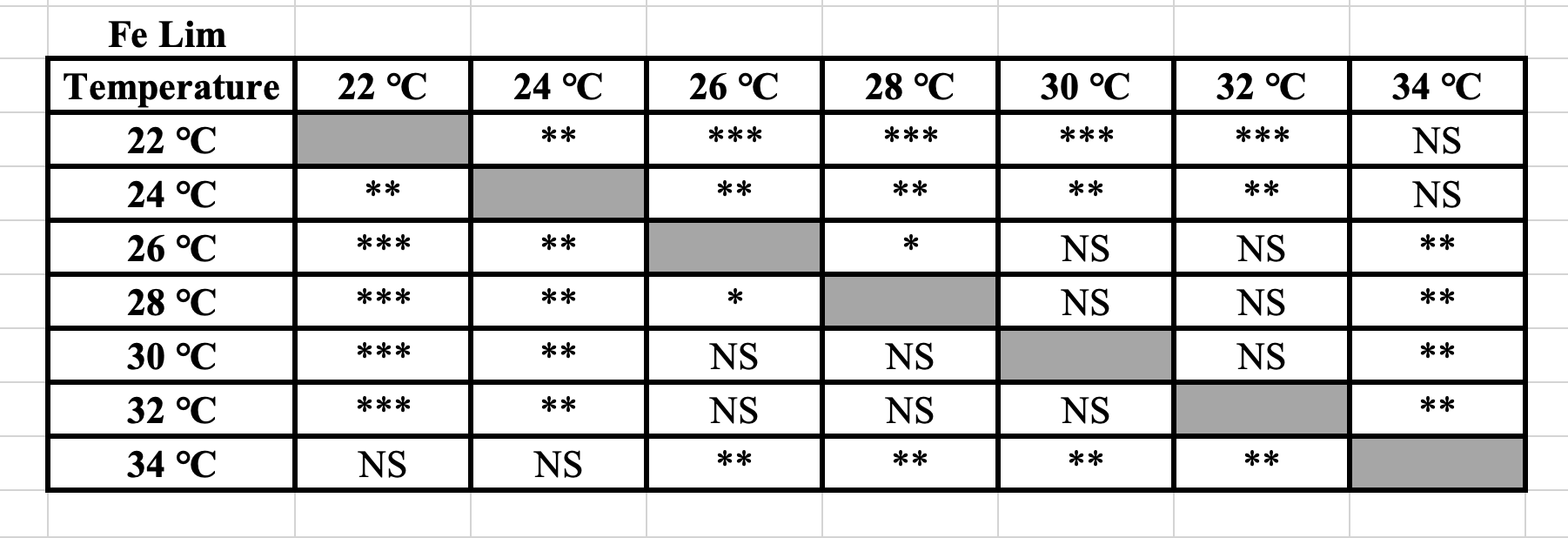
**

Asterisks indicate significance between temperatures: *** = *p* < 0.001, ** = *p* < 0.01, * = *p* < 0.05, and NS = not significant.

**Table 6.** Statistical significance table of the growth rates of phosphorus limited cultures at various temperatures.

**
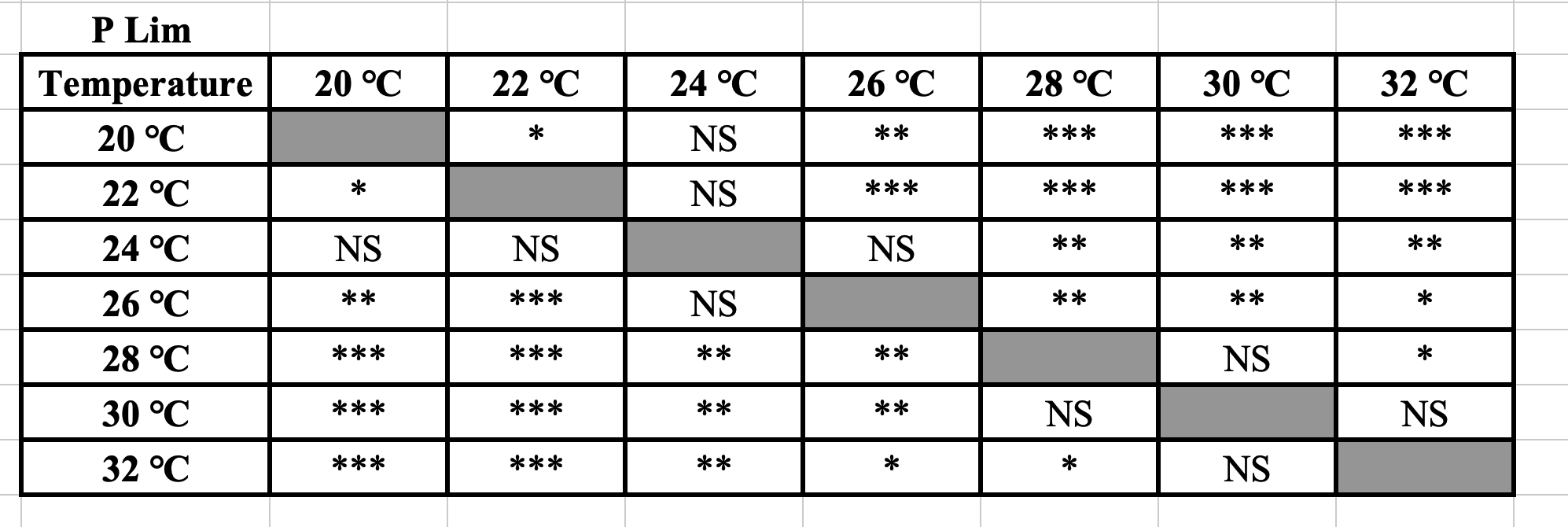
**

Asterisks indicate significance between temperatures: *** = *p* < 0.001, ** = *p* < 0.01, * = *p* < 0.05, and NS = not significant.

***Carbon Fixation Significance Tables***

**Table 7.** Statistical significance table of the carbon fixation rates of replete cultures at various temperatures.


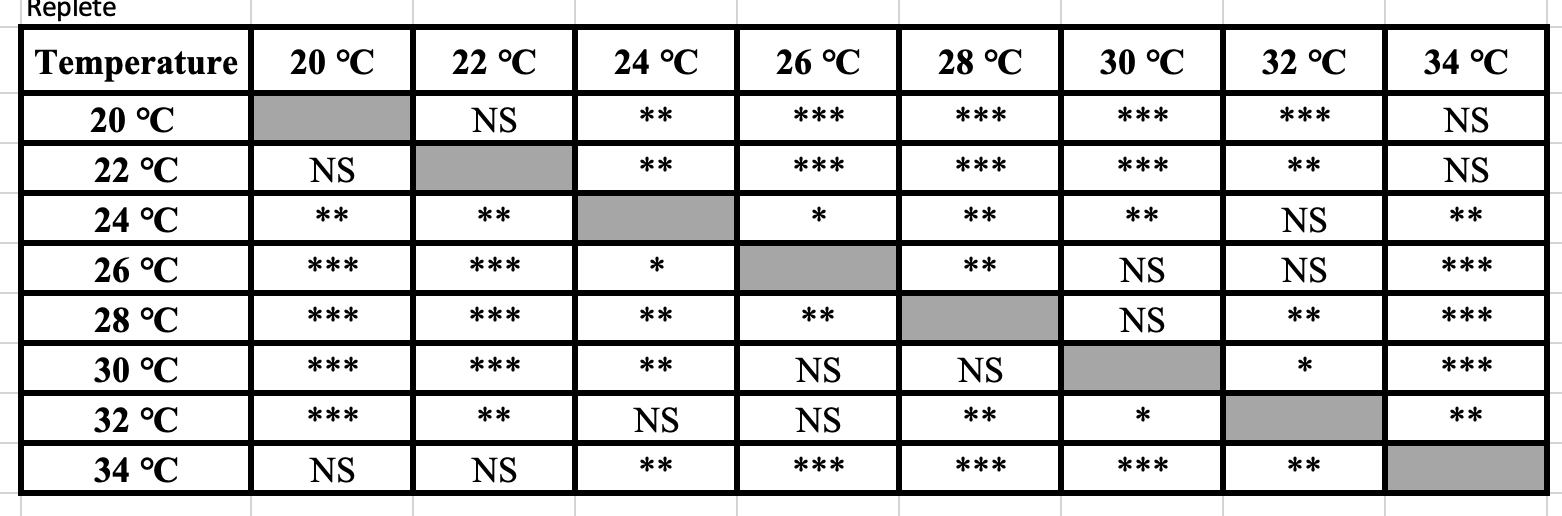


Asterisks indicate significance between temperatures: *** = *p* < 0.001, ** = *p* < 0.01, * = *p* < 0.05, and NS = not significant.

**Table 8.** Statistical significance table of the carbon fixation rates of iron limited cultures at various temperatures.

**
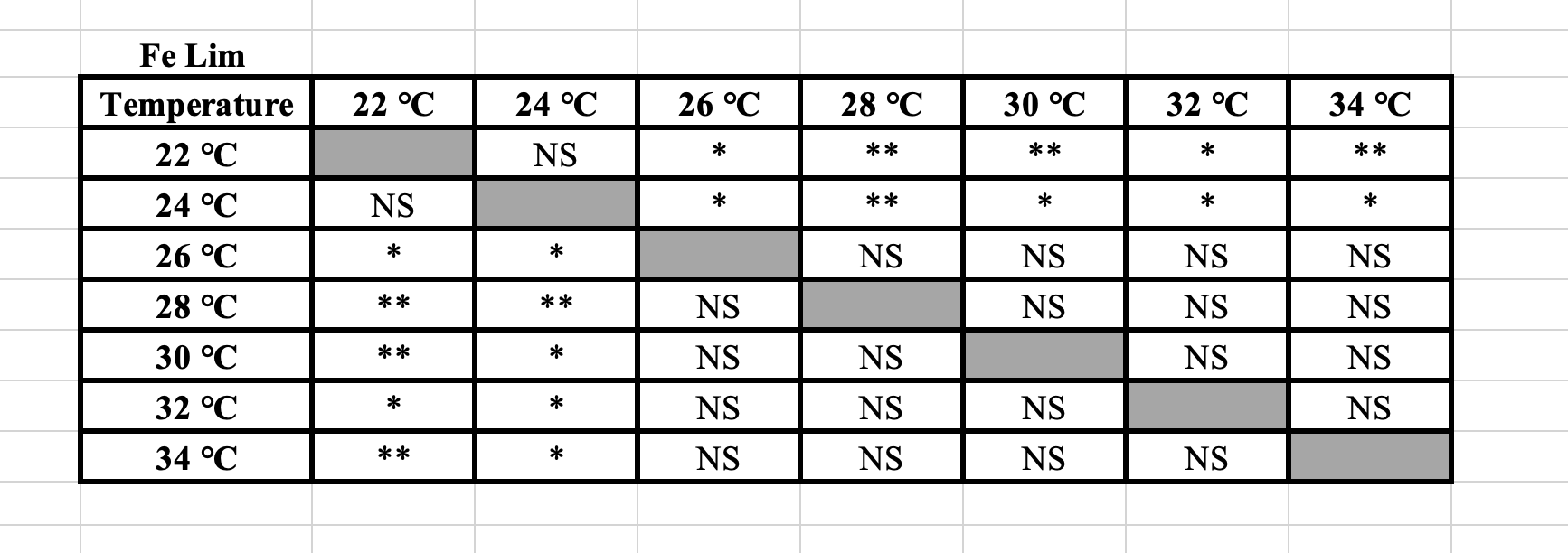
**

Asterisks indicate significance between temperatures: *** = *p* < 0.001, ** = *p* < 0.01, * = *p* < 0.05, and NS = not significant.

**Table 9.** Statistical significance table of the carbon fixation rates of phosphorus limited cultures at various temperatures.

**
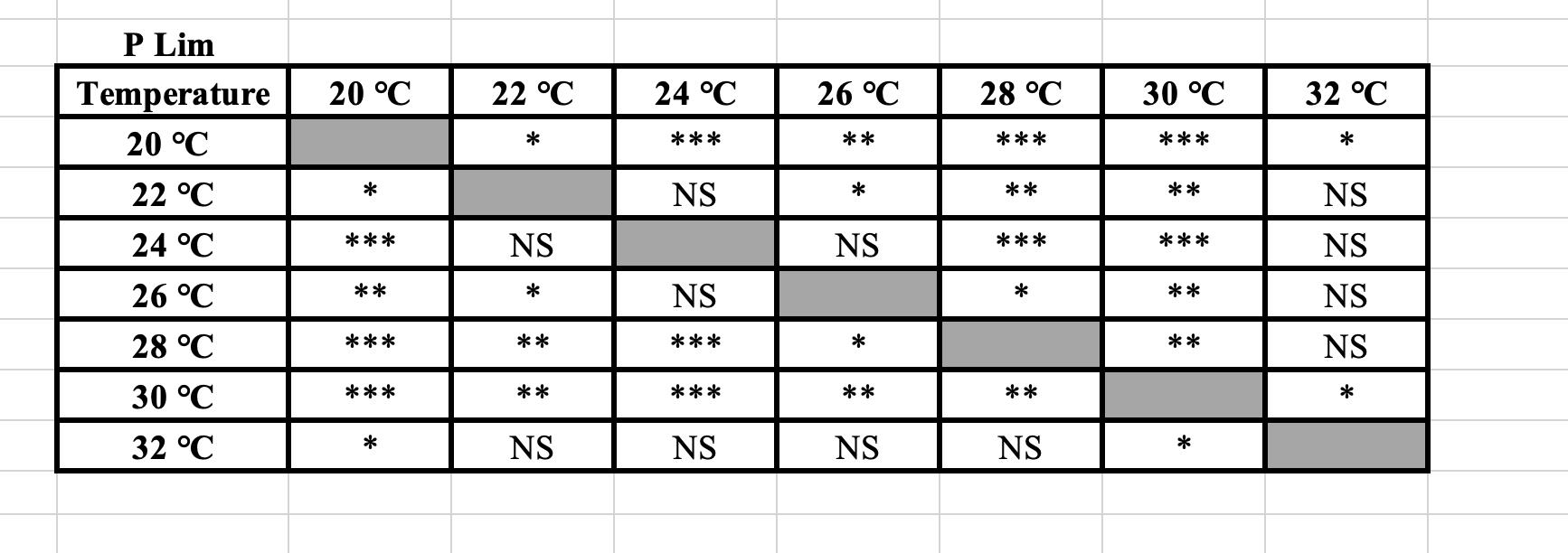
**

Asterisks indicate significance between temperatures: *** = *p* < 0.001, ** = *p* < 0.01, * = *p* < 0.05, and NS = not significant.

***Nitrogen Fixation Significance Tables***

**Table 10.** Statistical significance table of the nitrogen fixation rates of replete cultures at various temperatures.

**
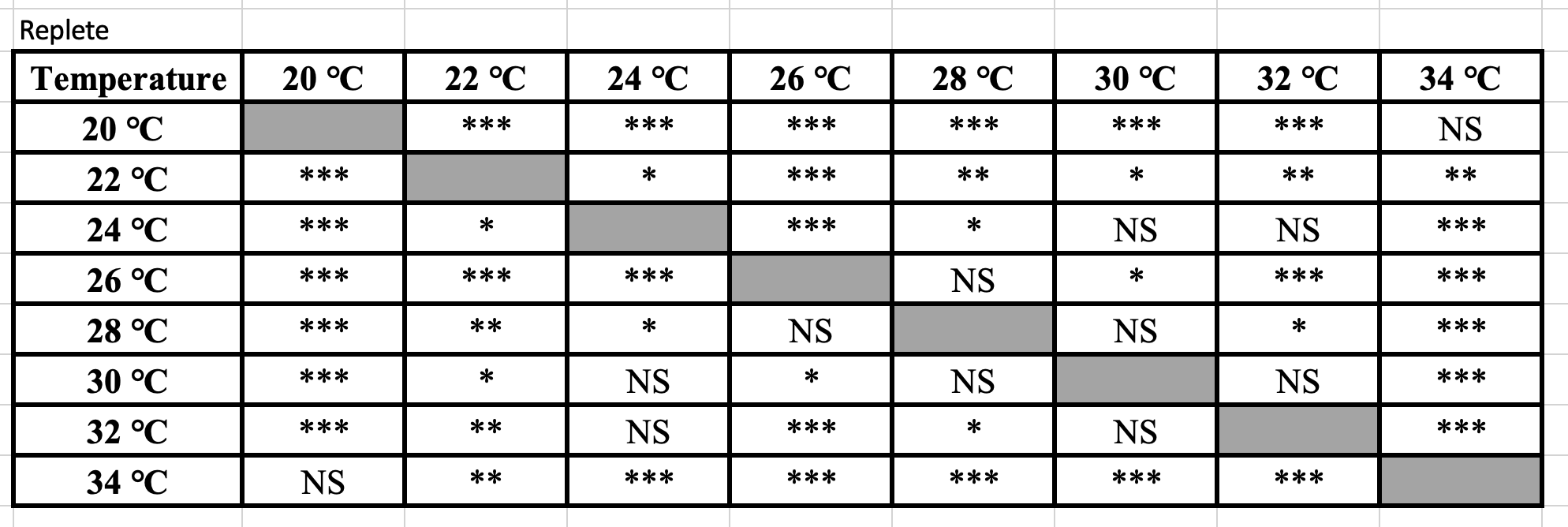
**

Asterisks indicate significance between temperatures: *** = *p* < 0.001, ** = *p* < 0.01, * = *p* < 0.05, and NS = not significant.

**Table 11.** Statistical significance table of the nitrogen fixation rates of iron limited cultures at various temperatures.

**
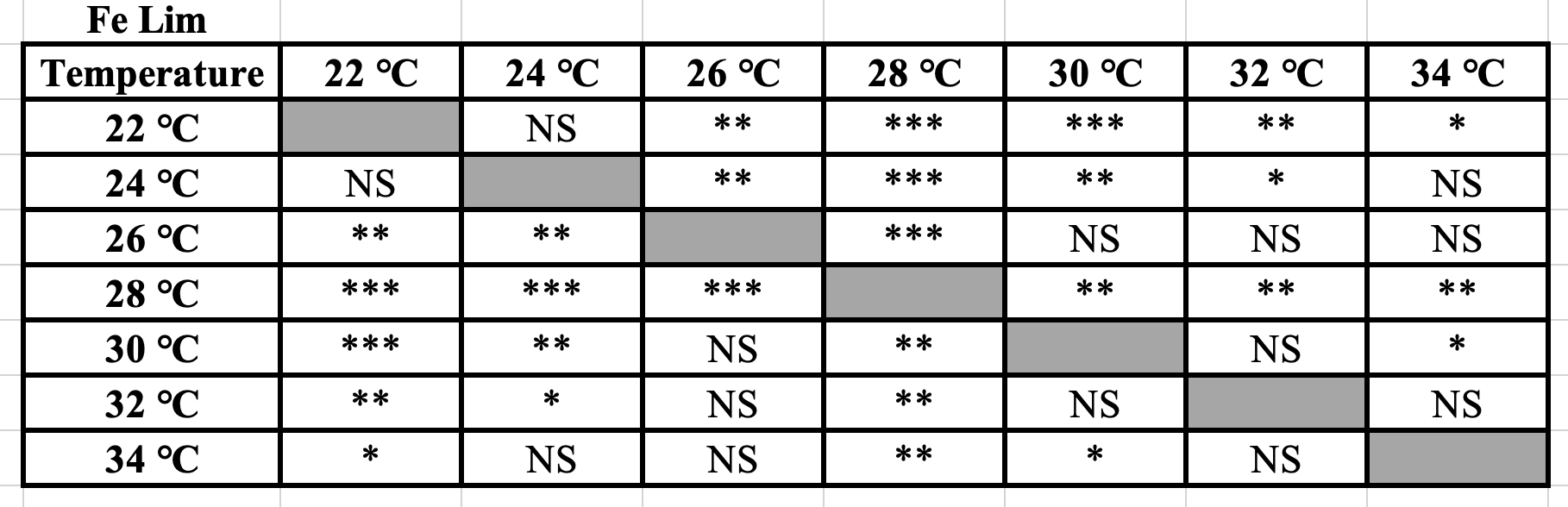
**

Asterisks indicate significance between temperatures: *** = *p* < 0.001, ** = *p* < 0.01, * = *p* < 0.05, and NS = not significant.

**Table 12.** Statistical significance table of the nitrogen fixation rates of phosphorus limited cultures at various temperatures.

**
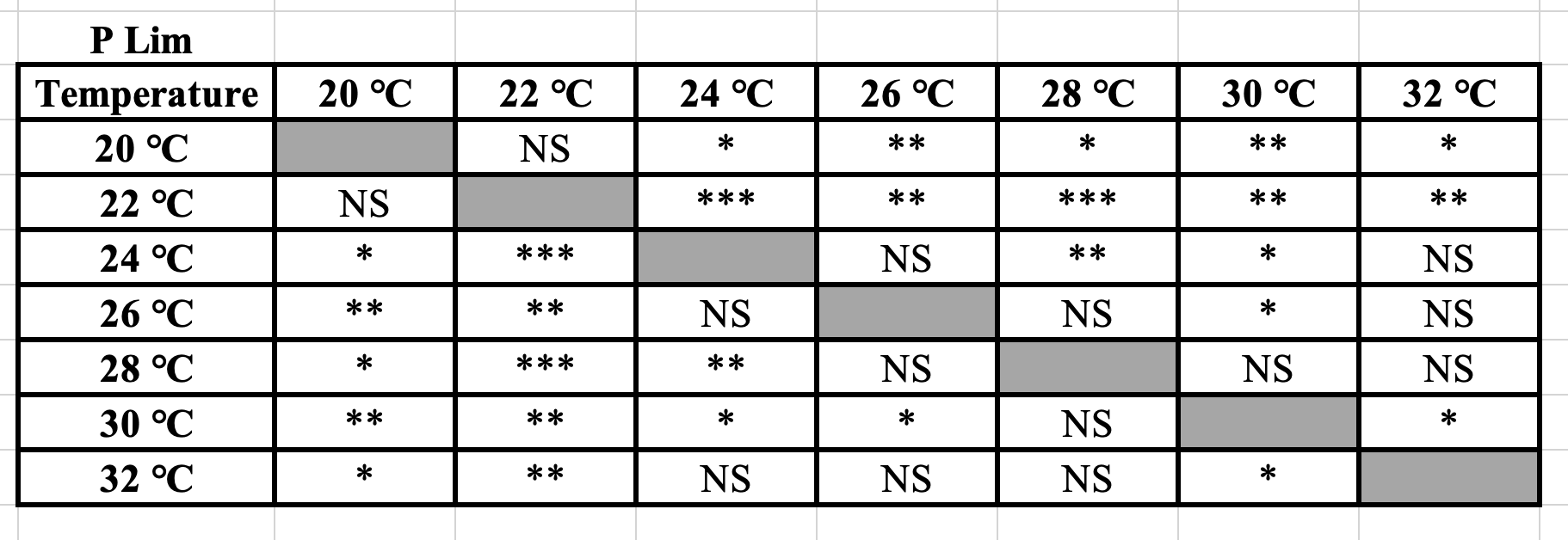
**

## Asterisks indicate significance between temperatures: *** = *p* < 0.001, ** = *p* < 0.01, * = *p* < 0.05, and NS = not significant.
